# Supplementary material for: Disentangling the effects of sex, life history and genetic background in Atlantic salmon: growth, heart and liver under common garden conditions
Source: R Soc Open Sci. 2020 Oct 7;7(10):200811. doi: 10.1098/rsos.200811 (PMC7657880; doi:10.1098/rsos.200811)
Supplement: Supplementary Growth and internal.docx [file rsos200811supp10.docx]

***Supplementary Material***

***Disentangling the effects of environment and genetics in Atlantic salmon: growth, heart and liver under common garden conditions***

William B Perry ^1^, Monica F Solberg ^2^, Christopher Brodie ^3^, Angela C Medina ^4^, Kirthana G Pillay ^1^, Anna Egerton ^1^, Alison Harvey ^2^, Simon Creer ^1^, Martin Llewellyn ^5^, Martin Taylor ^6^, Gary Carvalho ^1^, Kevin Glover ^2,7^

*1 = Molecular Ecology and Fisheries Genetics Laboratory, School of Biological Sciences, Bangor University, Bangor, Gwynedd LL57 2UW, UK.* [*w.perry@bangor.ac.uk*](mailto:w.perry@bangor.ac.uk)

*2 = Population genetics research group, Institute of Marine Research, P.O. Box 1870, Nordnes, NO-5817, Bergen, Norway.*

*3 =* *Mariani Molecular Ecology Laboratory, School of Natural Sciences and Psychology, Liverpool John Moores University, Liverpool, L3 5UX, UK.*

*4 =* *School of Microbiology, Food Science & Technology Building University College Cork, Cork, T12 TP07, Ireland.*

*5 = Institute of Biodiversity, Animal Health & Comparative Medicine, University of Glasgow, Glasgow, G12 8QQ, UK.*

*6 = School of Biological Sciences, University of East Anglia, Norwich, NR4 7TJ, UK.*

*7 = Institute of Biology, University of Bergen, Bergen, Norway*


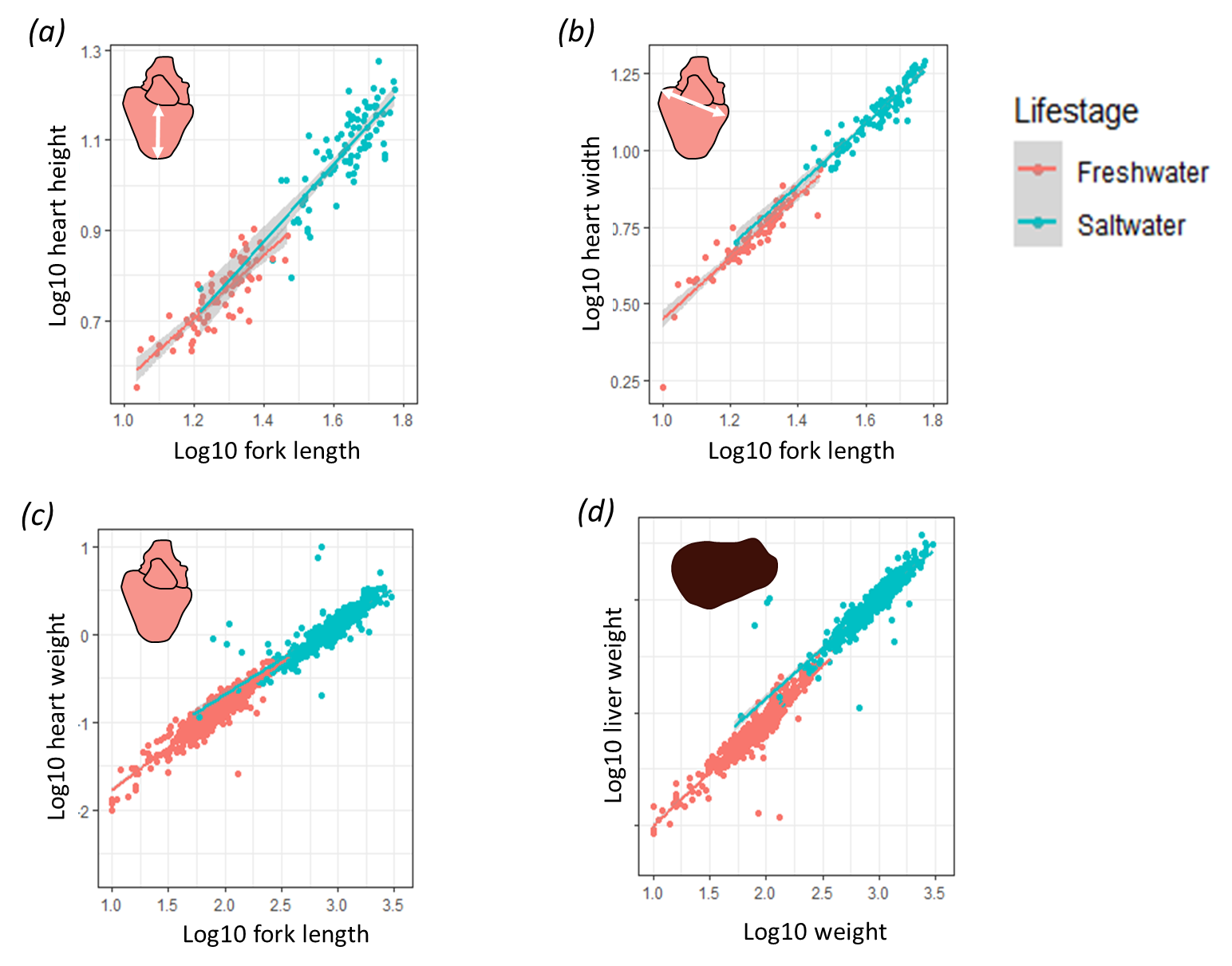


Supplementary figure 1 Linear regressions between log10 transformed (a) heart height, (b) heart width, (c) heart weight, (d) liver weight and log10 transformed fork length/log10 transformed body weight. Life stages are separated here, with independent regression lines, however, for the creation of adjusted measurements used in the study, one regression was used between both freshwater and saltwater life stages.


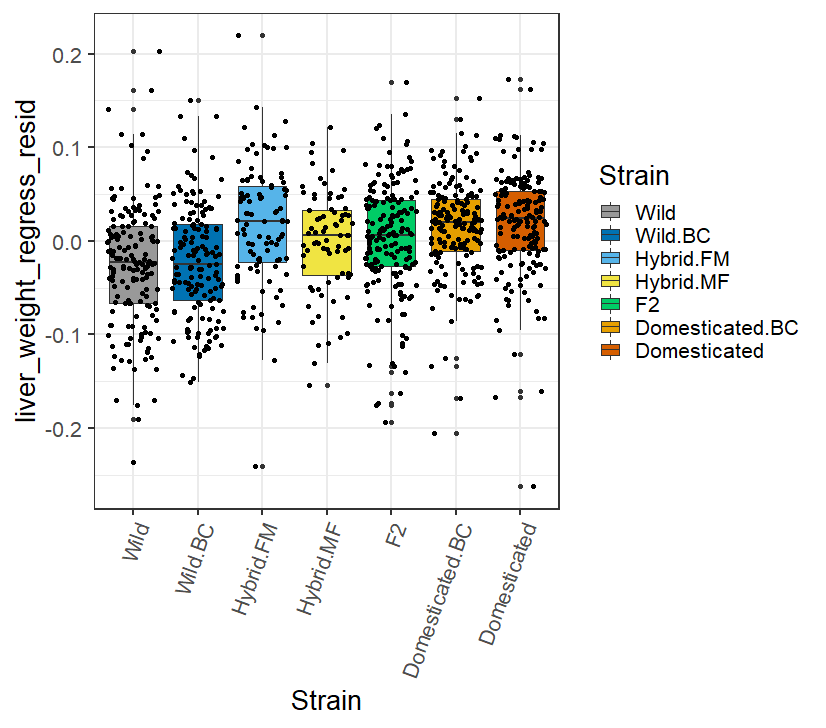


Supplementary figure 2 Body weight adjusted liver weight between the seven experimental strains. No significant differences were found between groups in the linear mixed effect models.

Supplementary table 1 Pairwise differences in mean log10 transformed wet body weight (g) between the seven experimental strains, with significant (p < 0.03) contrasts between means highlighted in bold. Results are based on freshwater and saltwater body weight combined.


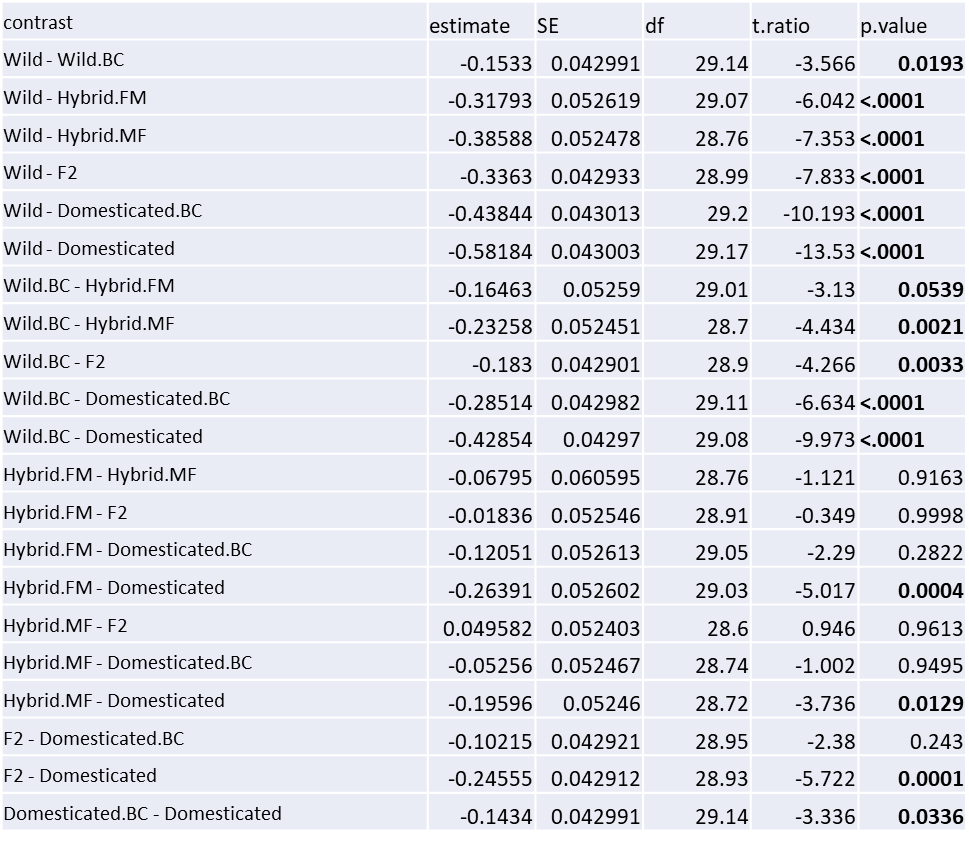


Supplementary table 2 Pairwise differences in log10 transformed mean wet body weight (g) between sexes in the seven experimental strains, with significant (p < 0.001) contrasts between means highlighted in bold. Results are based on freshwater and saltwater body weight combined.


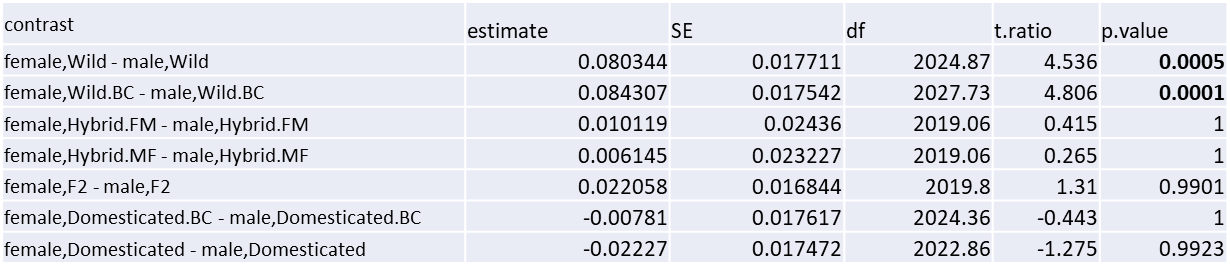


Supplementary table 3 p values from the linear mixed effect model for the differences in log10 mean weight (g) between strains, in freshwater and saltwater life stages, along with the trend in log10 mean weight between those strains. All trends between strains were consistent between life stages. Significance of trends, as assessed by p values (P < 0.05), were not consistent between life stages, however. Yellow highlighted trends show that the trend became significant in the saltwater life stage, but were not significant in the freshwater life stage. Red highlighted trends show that the trend lost significance in the saltwater life stage, but was significant in the freshwater life stage.


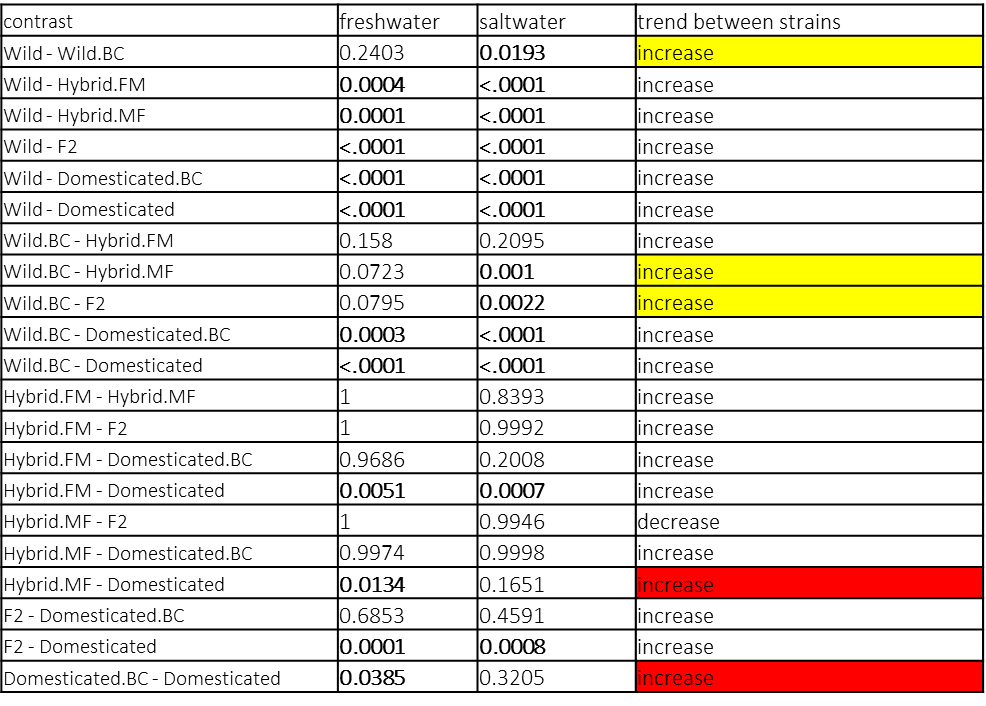


Once pairwise differences between strains were partitioned into life stages, 11/14 strains showed significant differences in fresh water, compared to 12/14 strains in the saltwater life stage (supplementary table 3). Some strains displayed significant differences in body weight in freshwater, but not in saltwater, and vice versa. Differences between 1) the wild backcross strain and the wild strain, 2) the wild backcross strain and the hybrid FM strain and 3) the wild backcross strain and the F2 strain, went from being non-significant in the fresh water life stage measurement (P > 0.05) to significantly different in the saltwater life stage measurement (supplementary table 3). Alternatively, differences between the domesticated strain and the hybrid MF and domesticated backcross strain went from being significant in freshwater to non-significant (p > 0.05) in the saltwater life stage (supplementary table 3). The difference in significance demonstrates that in the freshwater life stage, the increases in log10 body weight seen with increased domesticated strain (up to 50 %) are accentuated to the point of significance, which is not seen in salt water. The opposite is seen with strains that have over 50 % domesticated strain, with the significant increases in log10 body weight minimised in the saltwater life stage, in comparison to the freshwater. Variation in growth due to family background (S.D. = 0.07) and tank (S.D. = 0.02) was detected and controlled for as random factors in the linear mixed effect model. Differences between strains in the fresh water and salt water life stage are likely due to domesticated saltwater fish reaching a maximum size for the tank and feed available, whereas saltwater wild fish are able to carry on growing to the same capacity. Additionally, fresh water domesticated fish are able to grow more rapidly than fresh water wild fish.

Supplementary table 4 Estimated marginal means (emmean) for log10 wet body weight (g), per strain, broken down by life stage, along with corresponding standard deviation (sd), standard error (SE), degrees of freedom (df) and upper (upper.CL) and lower confidence limits (lower.CL).
